# Throughput Fairness Enhancement Using Differentiated Channel Access in Heterogeneous Sensor Networks

**DOI:** 10.3390/s110706629

**Published:** 2011-06-27

**Authors:** Eui-Jik Kim, Taeshik Shon, James Jong Hyuk Park, Young-Sik Jeong

**Affiliations:** 1 Department of Electrical Engineering, Korea University, Anam-dong 5-ga, Seongbuk-gu, Seoul, 136-713, Korea; E-Mail: woozic@korea.ac.kr; 2 Ajou University, San 5, Woncheon-dong, Yeongtong-gu, Suwon, 443-749, Korea; E-Mail: 743zh2k@gmail.com; 3 Seoul National University of Science and Technology, 172, Gongreung 2-dong, Nowon, Seoul, 139-743, Korea; E-Mail: parkjonghyuk1@hotmail.com; 4 Wonkwang University, 344-2 Shinyong-dong, Iksan, Jeonbuk 570-749, Korea

**Keywords:** throughput fairness, CSMA/CA, heterogeneous sensor networks

## Abstract

Nowadays, with wireless sensor networks (WSNs) being widely applied to diverse applications, heterogeneous sensor networks (HSNs), which can simultaneously support multiple sensing tasks in a common sensor field, are being considered as the general form of WSN system deployment. In HSNs, each application generates data packets with a different size, thereby resulting in fairness issues in terms of the network performance. In this paper, we present the design and performance evaluation of a differentiated channel access scheme (abbreviated to DiffCA) to resolve the fairness problem in HSNs. DiffCA achieves fair performance among the application groups by providing each node with an additional backoff counter, whose value varies according to the size of the packets. A mathematical model based on the discrete time Markov chain is presented and is analyzed to measure the performance of DiffCA. The numerical results show that the performance degradation of disadvantaged application groups can be effectively compensated for by DiffCA. Simulation results are given to verify the accuracy of the numerical model.

## 1. Introduction

Nowadays, wireless sensor networks (WSNs) are widely applied in diverse applications, e.g., habitat monitoring, healthcare, target tracking, battlefield surveillance, *etc.* [1–5]. Furthermore, it is commonly considered that one sensor network could be allocated one or more sensing tasks and serve several applications, simultaneously. We call this type of network a heterogeneous sensor network (HSN), in which one or more WSN subsystems coexist in one sensor field and forward the sensing data to a common sink node. An example of this would be the HSNs deployed in hospital buildings for applications such as a HVAC system, healthcare monitoring, and a security system. Consequently, the sensor nodes in this network gather various kinds of sensing data ranging from text-based information (e.g., temperature, humidity) to fixed image-based information (e.g., CCTV monitoring images). It can easily be inferred that the sizes of the data packets generated by each type of sensor differ significantly from each other. In other words, each node in the network generates and transmits data packets with different sizes depending on the task that it is in charge of.

Due to this salient feature of HSNs, particular services may suffer from unequal performance among the nodes. Considering contention-based carrier sense multiple access (CSMA) MAC, where the nodes carry out random backoff to avoid collisions and then sense the medium to access the channel, there necessarily exist advantaged and disadvantaged nodes. The former consist of those sensor nodes generating data with a large size such as CCTV still images, and the latter consist of those nodes transmitting packets with a relatively small size, that is text-based data such as the numerical values of temperature, humidity, and luminance measurements. While the nodes generating large data occupy the channel and transmit their data packets, those generating small data cannot avoid performing backoff until the channel becomes idle. Even if they seize the opportunity to occupy the channel, their transmission may be over shortly, and the transmission of large data packets will start again. Thus, the performance of small data generating nodes can be severely degraded. In the instance of hospital buildings mentioned above, the HVAC system might even be disabled, because of the transmissions of nodes performing other tasks (e.g., healthcare monitoring and security system).

In the literature [6–8], various attempts to solve the fairness problem using transmission control protocols for network congestion mitigation have been reported. ESRT [6], which is a centralized transmission control scheme, allocates the transmission rates to the sensor nodes such that an application-specific number of sensor readings are received at the sink, which prevents network congestion. Meanwhile, Fusion [7] and FACC [8] take a distributed transmission control approach. Fusion [7] uses the queue length to measure the level of congestion and integrates three techniques: hop-by-hop flow control, rate control, and prioritized MAC. With this combination, it achieves better fairness with heavy loads. FACC [8] adjusts the sending rate of each flow as early as possible and saves the precious resources at those nodes close to the sink. It categorizes all intermediate sensor nodes into near-source nodes and near-sink nodes and then assigns an appropriate rate to the near-source nodes to save energy and avoid congestion at the same time. However, these protocols commonly aim to achieve fairness among the different flows in terms of bandwidth allocation. None of the above transmission protocols was designed with the fairness of the application or service itself in mind.

In this paper, we focus on the fairness problem among the node groups performing different sensing tasks and aim to achieve fair levels of performance, which are sufficient to serve each application within the HSN. For this purpose, we propose a novel differentiated channel access scheme (abbreviated to DiffCA) that gives the nodes approximately fair channel access opportunities. By using DiffCA, the nodes are given additional backoff counters with different values according to their packet size. When the channel is sensed to be busy, the nodes conduct the backoff procedure with the given backoff counter and then try to access the channel again. The mathematical model is based on the discrete-time Markov chain in which each component of an element in state space represents the situation of the head packet in the queue of a node. By analyzing the Markov chain, we obtain the transmission probability, the collision probability, and the saturation throughput. In what follows, we describe the design and performance of our proposed solution in detail.

## 2. HSN Model

**Figure 1.**
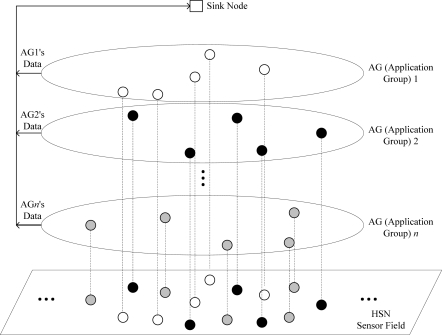
Heterogeneous sensor network (HSN) model.

We assume a general network model of HSNs and Figure 1 shows its conceptual topology. In the figure, in order to support one or more WSN applications simultaneously, the HSN includes several application groups (AGs) which correspond to groups of nodes that are responsible for a common sensing task.

It would be expected that the packets generated from nodes belonging to the same AG are nearly equal in size. In terms of the network layer, a star topology is assumed, where each node in the network transmits its sensing data packet to a sink node directly. The link layer is assumed to use the IEEE 802.15.4 MAC based protocol, which is a typical contention-based CSMA solution [9]. Without regard for the characteristics of the AG, the sensor nodes share the same wireless medium. Therefore, they have the same opportunity to occupy the channel, which results in the following problem: when a node generating packets of a relatively large size seizes the channel, the nodes with small packet sizes sense the busy channel and re-conduct random backoff with a 2-times increased backoff selection range. It is certain that the transmission of nodes with small sized packets becomes more difficult over time and their performance can be severely degraded. Therefore, a countermeasure to compensate for this performance degradation imposed by the difference in the packet size is needed.

## 3. Design of Differentiated Channel Access (DiffCA) Scheme

Our work focuses on the service feasibility of each application within an HSN, which is different from the traditional notions of fairness and congestion control. For a particular WSN service to work without any problems, the sensor nodes belonging to each AG should enjoy a sufficient level of network throughput (or data rate) to deliver their sensing information. As mentioned above, in legacy IEEE 802.15.4 systems, the difference in the packet size causes advantaged and disadvantaged groups for the throughput context; in the worst case, this imbalance might always hinder or disable the service of the most disadvantaged group. Thus, in order to guarantee the service feasibilities for all of the services that the HSN maintains, DiffCA aims to provide fair network throughput among the different AGs, which can be achieved by differentiating the transmission probabilities depending on the size of the generated data packets.

DiffCA is a modified version of the slotted CSMA/CA protocol operating in an IEEE 802.15.4 beacon-enabled network. Let us take a brief look at the CSMA/CA protocol of legacy IEEE 802.15.4 systems, in which the sink node divides its channel time into superframes. Each superframe consists of an active portion and an optional inactive portion. In the contention access period (CAP) within the active portion, the nodes transmit the data packets in a contention manner [9,10]. In this paper, we only consider this CAP duration and analyze DiffCA under saturation conditions.

The basic concept of DiffCA is to give priority to the disadvantaged nodes in terms of channel access, namely those nodes with small sized packets, by differentiating the backoff counters of the CSMA/CA protocol. More specifically, when the channel is sensed to be busy at the first clear channel assessment (CCA) procedure, the node re-conducts an additional backoff instead of proceeding to the second CCA. Giving a higher transmission priority to small packets seems to be counter-intuitive from the quality of service (QoS) perspective for individual traffic. However, this approach can provide a reasonable solution, since DiffCA seeks to achieve fairness in terms of the service feasibilities, rather than the traffic itself. Note that DiffCA maintains the relatively fair service feasibility for all of the AGs in the HSN, which is distinct from the notion of the absolute QoS requirement of the specific application. The additional backoff counter (ABC) of node *k* is determined in proportion to its packet size, which can be represented as follows:

(1) $$ABC[k]=(T_{H}+T_{E[L_{k}(i)]}+\gamma +T_{ACK}+t_{ACK})\times (\text{unit backoff period}/\text{second})$$

where *T_H_*, *T*_*E*[*L_k_*(*i*)]_, *γ*, *T_ACK_*, and *t_ACK_* denote the time required to transmit the header (including the MAC and PHY headers), the average time required to transmit the payload of the packet which node *k* generates, the propagation delay, the time required to transmit an ACK, and the time required to receive the first bit of the ACK, respectively.

**Figure 2.**
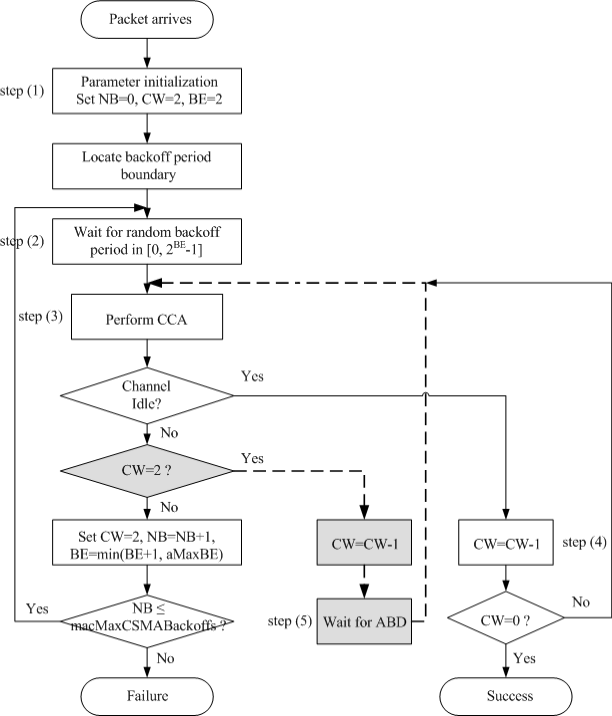
Operation of the differentiated channel access scheme.

It can be intuitively deduced that a smaller number of additional backoff counters is given to those nodes with small sized packets and, therefore, they can obtain access to the channel earlier than those nodes with large sized packets. Note that additional backoff occurs only in the case where the channel is sensed to be busy. DiffCA considers the contention level of the network. Under low contention, DiffCA is not effective, since all of the application systems can function normally without it. However, under high contention where the wireless channel is likely to be busy, DiffCA may effectively guarantee an acceptable degree of performance for disadvantaged nodes for them to achieve normal operation. The flowchart of Figure 2 describes the operation of DiffCA in detail.

The gray boxes and dotted lines depict the modifications made to the legacy IEEE 802.15.4 CSMA/CA in DiffCA. In the figure, the number of backoffs (NB) denotes the number of times that the algorithm is required to backoff due to the unavailability of the medium during channel assessment. The contention window (CW) is the number of backoff periods that need to be clear of channel activity before the packet transmission can begin. Finally, BE denotes the backoff exponent, which is related to the number of backoff periods that a node should wait before attempting to assess the channel. In step (1), three variables NB, CW and BE are set to zero, 2, and 2, respectively. Then, the algorithm locates the boundary of the next backoff period. In step (2), a random waiting time in the range of [0, 2^BE^ – 1] is generated. When this time is over, the node will proceed to step (3) and perform the first CCA to see whether the medium is idle. If the channel is idle, the procedure proceeds to step (4) and the channel is reviewed once more. When the value of CW becomes zero, the packet transmission may begin, provided that the remaining number of backoff periods in the current superframe suffices to handle both the packet and the subsequent acknowledgment. At the first CCA, if the channel is sensed to be busy, the node performs the additional backoff required by DiffCA, whose counter value is calculated by Equation (1) [step (5)]. When this additional waiting time is over, the node performs the second CCA in common with the legacy IEEE 802.15.4 CSMA/CA algorithm [step (6)]. The residual procedures are the same as those of the legacy algorithm.

## 4. Analytical Model

For convenience of analysis, we use the HSN model described in Section 2, which includes several AGs, and assume that those nodes belonging to the same AG generate data packets with the same size. Moreover, we consider only the packet transmission of each individual sensor node in the network to derive the performance metrics in the MAC-layer, such as the transmission probability, collision probability and throughput, which is independent of the specific network topology that is formed. This approach is commonly used to evaluate the network performance in the literature [10–13]. To analyze the proposed scheme, we introduce the following three random variables for a given node in the *g* th AG. Let *nb*(*g*,*t*), *cw*(*g*,*t*), and *bc*(*g*,*t*) be the stochastic processes representing the value of NB, CW, and the value of the backoff counter, respectively, at time *t*. Note that NB represents the backoff stage within the range of [0, *m*], where *m* = macMaxCSMABackoffs whose default value is 4 in the IEEE 802.15.4 standard [9]. Furthermore, throughout this paper, ‘*g*’ means the *g* th AG and gives the different priorities taking integer values in [0, G], where (G + 1) is the number of AGs generating packets with different sizes in the network. The process {(*nb*(*g*,*t*),*cw*(*g*,*t*),*bc*(*g*,*t*))} forms a multi-dimensional Markov process defining the state of the packet at the backoff unit boundaries. Since we assume that each node has its own priority according to the AG it belongs to, which does not change, each of the processes, *nb*(*g*,*t*), *cw*(*g*,*t*), *bc*(*g*,*t*), can be written simply as *nb*(*t*), *cw*(*t*), *bc*(*t*). Then, the corresponding state space is denoted as Ω = {(*nb*(*t*),*cw*(*t*),*bc*(*t*))}, where 0≤*nb*(*t*)≤*m*+1, 0≤*cw*(*t*)≤2, *W*_0_ = 2*^BE^*, *W_i_* = 2*^i^W*_0_, and *i* = 0,…, *m*. *nb*(*t*), *cw*(*t*) and *bc*(*t*) are integers and the value of *bc*(*t*) differs according to the value of *cw*(*t*):

(2) $$bc(t)=\{\begin{array}{lll} 0∼W_{i}–1, & i\in [0,m], & \text{if }cw(t)=1\\ ABC[g], & g\in [0,G], & \text{if }cw(t)=2 \end{array}$$

*ABC*[*g*] is the additional backoff counter (ABC) of the *g* th AG, which is given by:

(3) $$ABC[g]=(T_{H}+T_{E[L_{g}(i)]}+\gamma +T_{ACK}+t_{ACK})\times (\text{unit backoff period}/\text{second})$$

where *T_H_*, *T*_*E*[*L*_*g*_(*i*)]_, *γ*, *T_ACK_*, and *t_ACK_* denote the time required to transmit the header (including the MAC and PHY headers), the average time required to transmit the payload of the packet which AG *g* generates, the propagation delay, the time required to transmit an ACK, and the time required to receive the first bit of the ACK, respectively.

**Figure 3.**
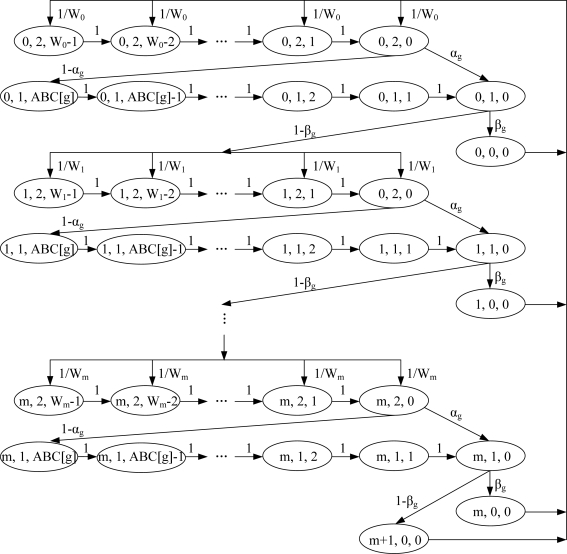
Markov chain model

The state transition diagram of these states is illustrated in Figure 3. For the simplicity of the notations, we use the transition probabilities *P*(*i, j, k* − 1|*i*, *j*, *k*) instead of *P*(*nb* (*t* + 1) = *i*, *cw* (*t* + 1) = *j*, *bc* (*t* + 1) = *k* − 1| *nb* (*t*) = *i*, *cw* (*t*) = *j*, *bc* (*t*) = (*k*). We assume that the total number of nodes is *n*, which are composed of *n_l_*, *l*∈[0, *G*] nodes in AG *l*. Furthermore, we assume that each of them always has a packet ready to be transmitted. The purpose of the assumption made for this saturation condition is to compare the relative performances among the AGs, rather than the real estimated values for the network throughput, which are likely to vary depending on the experimental environment. The existing analytical models [10–13] also consider the nodes operating in the saturation mode for the same reason. Moreover, DiffCA provides the solution to the performance degradation problem of the disadvantaged node group under high contention. Thus, intuitively, the analysis in the saturation condition is suitable as a comparison with the legacy scheme. Then, the one-step transition probabilities are given as follows:

(4) $$P(0,2,k|i,0,0)=1/W_{0},\;\;i\in [0,m],k\in [0,W_{0}–1],$$

(5) $$P(0,2,k|m+1,0,0)=1/W_{0},\;\;k\in [0,W_{0}–1],$$

(6) $$P(i,2,k–1|i,2,k)=1,\;\;\;i\in [0,m],k\in [1,W_{i}–1],$$

(7) P(i,1,k–1|i,1,k)=1,   i∈[0,m],k∈[1,ABC[g]],

(8) $$P(i,1,0|i,2,0)=\alpha_{g},\;\;i\in [0,m],$$

(9) $$P(i,0,0|i,1,0)=\beta_{g},\;\;\;i\in [0,m],$$

(10) $$P(i,1,ABC[g]|i,2,0)=1–\alpha_{g},\;\;i\in [0,m],$$

(11) $$P(i+1,2,k|i,1,0)=(1–\beta_{g})/W_{i+1},\;\;\;i\in [0,m–1],k\in [0,W_{i+1}–1],$$

(12) $$P(m+1,0,0|m,1,0)=1–\beta_{g}$$

Equation (4) represents the probability that a new packet transmitted following a successful channel access starts with backoff stage 0 and, thus, the backoff counter is initially uniformly chosen in the range of [0, *W*_0_, − 1]. Therefore, this probability is 1/*W*_0_. Note that the random backoff period always precedes packet transmission, regardless of whether the packet to be transmitted has just arrived or it is a packet that could not be transmitted in the previous attempt due to collision. Equation (5) is the probability in the case where the previous attempt to transmit a packet ended in failure and the device begins to perform the algorithm again for a new packet, which is equal to 1/*W_0_*. Our scheme has two situations where the backoff counter can be decreased. In the first case, Equation (6) is the probability that the backoff counter is decreased by one at the beginning of each time slot before the first CCA in each backoff stage. In the second case, Equation (7) is the probability that the backoff counter is decreased by one at the beginning of each time slot before the second CCA in the case where the channel is sensed to be busy at the first CCA. Note that, in Equation (7), the value of the backoff counter, *k*, is initially *ABC*[*g*], which enables differentiated channel access according to the packet size of each AG. Equation (8) is the probability α*_g_* that the CW value is decreased by one after an idle slot is detected during the first CCA procedure in each backoff stage. Equation (9) is the probability β*_g_* that the CW value is decreased by one after an idle slot is detected during the second CCA procedure in each backoff stage and this makes the node transmit the packet at the boundary of the next slot. Equation (10) is the probability 1–α*_g_* that the CW value is decreased by one after a busy slot is detected during the first CCA procedure in each backoff stage. The proposed scheme makes additional backoff counters of *ABC*[*g*] after the first CCA procedure operated in the busy channel condition. Therefore, the backoff counter is determined initially to be *ABC*[*g*]. Equation (11) is the probability that the device chooses another random backoff counter in the next backoff stage when it senses the channel to be busy in the second CCA procedure and, thus, at backoff stage *i+*1, the backoff counter is chosen uniformly in the range of [0,*W*_*i*+1_−1]. Thus, this probability is given by (1–β*_g_*)/*W*_*i*+1_. Finally, Equation (12) is the probability that the last attempt to transmit the packet failed and the value of the backoff stage exceeds *m*, where *m* = macMaxCSMABackoffs. In this case, the node will prepare a new transmission of the next packet which is located at the boundary of the queue. Note that all of the states in Ω are positive recurrences and the system is stable. Therefore, there exist stationary probabilities {*b_i,j,k_* } of the discrete-time Markov chain which is defined by:

(13) $$\begin{array}{l} b_{i,j,k}=\underset{t\to \infty }{\text{lim}}P\{nb(t)=i,cw(t)=j,bc(t)=k\},\;\;\;i\in [0,m+1],j\in [0,2]\;\text{and}\\ \;k=\{\begin{array}{lll} 0∼W_{i}–1, & i\in [0,m], & \text{if }j=1\\ ABC[g], & g\in [0,G], & \text{if }j=2 \end{array} \end{array}$$

Let **b** be the stationary vector, *i.e.*, **b** = (*b*_0,0,0_, *b*_0,1,0_, …, *b_m_*_+1,0,0_). Then it satisfies the following:

(14) bP=b and be=1,

where **e** is the column vector whose components consist of 1 and **P** is the transition probability matrix when Ω is ordered lexicographically. By using the first part of Equation (14), we obtain the following relations between stationary probabilities:

(15) $$\begin{array}{c} b_{i,0,0}=b_{0,0,0}{\left(1–\beta_{g}\right)}^{i},\;\;\;i\in [0,m],\\ b_{i,2,k}=b_{0,0,0}\frac{\left(W_{i}–k\right)}{W_{i}}\frac{{\left(1–\beta_{g}\right)}^{i}}{\beta_{g}},\;\;i\in [0,m],k\in [0,W_{i}–1],\\ b_{i,1,k}=b_{0,0,0}\frac{(1–\alpha_{g}){\left(1–\beta_{g}\right)}^{i}}{\beta_{g}},\;\;i\in [0,m],k\in [1,ABC[g]],\\ b_{i,1,0}=b_{0,0,0}\frac{{\left(1–\beta_{g}\right)}^{i}}{\beta_{g}},\;\;i\in [0,m],\\ b_{m+1,0,0}=b_{0,0,0}\frac{{\left(1–\beta_{g}\right)}^{m+1}}{\beta_{g}}, \end{array}$$

By substituting Equation (15) into the second part of Equation (14), we obtain *b*_0,0,0_ as follows:

(16) $$b_{0,0,0}=2\beta_{g}/\{\sum_{i=0}^{m}(W_{0}2^{i}+1){\left(1–\beta_{g}\right)}^{i}+2(ABC[g](1–\alpha_{g})+1+\beta_{g})\sum_{i=0}^{m}{\left(1–\beta_{g}\right)}^{i}+2{\left(1–\beta_{g}\right)}^{m+1}\}$$

By substituting Equation (16) into each equation in Equation (15), we obtain the stationary probabilities {*b_i,j.k_*}. With these stationary probabilities, we find the probability that the sensor node transmits a packet at the boundary of a backoff period, which is denoted by τ. Let τ*_g_* be the probability that a sensor node in the AG *g* starts the transmission during a generic slot time. Then we have:

(17) $$\tau_{g}=\sum_{i=0}^{m}b_{i,0,0}$$

Since the node belongs to different AGs that have different elements such as *ABC*[*g*], α*_g_* and β*_g_*, the index *g* of τ is needed to differentiate the AGs. α*_g_* and β*_g_* mean the probabilities that the node senses that the channel is idle in the first and second CCA procedures, respectively. Also, the probability that the channel is idle at the end of the backoff counting is the probability that other $(n_{g}–1)+\sum_{i=0,i\neq g}^{G}n_{i}$ nodes are not transmitting. Assuming that the average slot times that are used for channel access for the nodes to transmit packets is *E*[*ABC*[*g*]], α*_g_* can be described by:

(18) $$\alpha_{g}=1–(1–{\left(1–\tau_{g}\right)}^{n_{g}–1}\prod_{i=0,i\neq g}^{G}{\left(1–\tau_{i}\right)}^{n_{i}})E[ABC[g]],\;\;g\in [0,G],n=\sum_{l=0}^{G}n_{l}.$$

(19) $$\begin{array}{ll} P(CW=1,\text{ idle channel})= & P(CW=1,\;\text{idle channel}|CW=2,\;\text{idle channel})\\ & \times P(CW=2,\;\text{idle channel})\\ & +P(CW=1,\;\text{idle channel}|CW=2,\;\text{busy channel})\\ & \times P(CW=2,\;\text{idle channel}). \end{array}$$

Because β*_g_* is dependent on α*_g_*, β*_g_* can be obtained from Equation (19). Therefore, β*_g_* can be described as:

(20) $$\begin{array}{l} \beta_{g}={\left(1–\tau_{g}\right)}^{n_{g}–1}\prod_{i=0,i\neq g}^{G}{\left(1–\tau_{i}\right)}^{n_{i}}+1–(1–{\left(1–\tau_{g}\right)}^{n_{g}–1}\prod_{i=0,i\neq g}^{G}{\left(1–\tau_{i}\right)}^{n_{i}})E[ABC[g]],\\ g\in [0,G],n=\sum_{l=0}^{G}n_{l}. \end{array}$$

## 5. Performance Analysis

### 5.1. Throughput

During the CCA procedure, the probability that the channel is idle is given by:

(21) $$P_{I}=\prod_{l=0}^{G}{\left(1–\tau_{l}\right)}^{n_{l}},\;\;\;\;n=\sum_{l=0}^{G}n_{l}.$$

Let *P_s_* and *P_s,g_* be the probabilities that successful transmissions are performed by a node in any AG and a node in the AG *g* in a time slot, respectively. Then, these probabilities are calculated as follows:

(22) $$P_{s}=\sum_{l=0}^{G}\frac{n_{l}\tau_{l}}{1–\tau_{l}}\prod_{h=0}^{G}{\left(1–\tau_{h}\right)}^{n_{h}}=P_{I}\sum_{l=0}^{G}\frac{n_{l}\tau_{l}}{1–\tau_{l}},\;\;n=\sum_{j=0}^{G}n_{j}\;\;and$$

(23) $$P_{s,g}=n_{g}\tau_{g}{\left(1–\tau_{g}\right)}^{n_{g}–1}\prod_{i=0,i\neq g}^{G}{\left(1–\tau_{i}\right)}^{n_{i}}=\frac{n_{g}\tau_{g}}{1–\tau_{g}}P_{I},\;\;g\in [0,G],n=\sum_{j=0}^{G}n_{j}.$$

Let *P_B_* be the probability that the channel is sensed to be busy in a time slot. Then, it is given by:

(24) $$P_{B}=1–P_{I}=1–\prod_{l=0}^{G}{\left(1–\tau_{l}\right)}^{n_{l}},\;\;n=\sum_{l=0}^{G}n_{l}.$$

Then *P_B_*–*P_s_* is the probability that the channel is sensed to be busy due to collisions that occurred from any AGs. From Equations (21–24), we can express the normalized throughput *S_g_* for the AG *g* as the following ratio:

(25) $$S_{g}=\frac{E(\text{payload transmitted in a slottime})}{E(\text{length of a slot time})}=\frac{P_{s,g}E[L_{g}(i)]}{P_{I}\delta +P_{s}T_{s}+(P_{B}–P_{s})T_{c}}$$

where *T_s_*, *T_c_*, δ, *L_g_*(*i*), and *E*[*L_g_*(*i*)] are the average time that the channel is sensed to be busy because of a successful transmission, the average time that the channel has a collision, the duration of an empty time slot, the payload size of the nodes in AG *g*, and the average payload size of AG *g*, respectively. Note that *T_s_* and *T_c_* are given by:

(26) $$T_{s}=T_{H}+T_{E(L)}+\gamma +T_{ACK}+t_{ACK}$$

and:

(27) $$T_{c}=T_{H}+T_{E(L*)}+\gamma ,$$

where *T_H_*, *T*_*E*(*L*)_, *γ*, *T_ACK_*, *t_ACK_*, L, L^*^ and *T*_*E*(L*)_ denote the time required to transmit the header (including the MAC and PHY headers), the time required to transmit the payload, the propagation delay, the time required to transmit an ACK, the time required to receive the first bit of the ACK from the receiver, the payload size of each node in the network, the payload size of each node during a collision, and the time required to transmit a payload with length *E*(L*), respectively. The symbol *E* stands for expectation.

### 5.2. Collision Probability

In this paper, for convenience of analysis, we assume that if a collision occurs, the packet is dropped and the next packet at the boundary of the queue will be prepared for transmission. Therefore, for the AG *g*, the probability for a packet to be dropped in a time slot is equal to the probability that there are at least two nodes which undergo collisions, which can be expressed as follows:

(28) $$\begin{array}{l} P_{d,g}=P_{c,g}=n_{g}\tau_{g}{\left(1–\tau_{g}\right)}^{n_{g}–1}(1–\prod_{l=0,l\neq g}^{G}{\left(1–\tau_{l}\right)}^{n_{l}})+\sum_{k=2}^{n_{g}}\left(\begin{array}{c} n_{g}\\ k \end{array}\right)\tau_{g}^{k}{\left(1–\tau_{g}\right)}^{n_{g}–k},\\ g\in [0,G],n=\sum_{j=0}^{G}n_{j}. \end{array}$$

## 6. Performance Evaluation

In this section, we present the performance of DiffCA and compare the analytical and simulation results to verify the accuracy of its numerical model. The simulations are conducted using the Matlab simulator, in which we only consider the simplified MAC-layer model of IEEE 802.15.4, exclusive of PHY-layer parameters such as the wireless channel noise. The parameters used in the numerical analysis and simulation refer to the BPSK mode listed in Table 1. Also, some assumptions are made for the purpose of simplifying the simulation and numerical analysis without sacrificing the comprehensive analysis of the model. The following assumptions can be made in the saturation mode which is considered in this paper. We assume that the size of the packets that the nodes within the same AG generate is constant and the ACK does not collide with the packets.

In order to verify the accuracy of the proposed model, a comparison of the throughputs with various numbers of nodes within each AG is presented in Table 2. As shown in Table 2, the simulation results are almost the same as those obtained in the numerical analysis, which are obtained with a 97.2% confidential rate (CR) which is calculated by the following equation:

(29) $$CR=\left(1–\left|\frac{E[S_{anal}–S_{sim}]}{E[s_{amal}]}\right|\right)\times 100\%$$

Since the differences between the analytical and simulation results are negligible, in the remaining figures, we present the results of the numerical analysis only.

**Figure 4.**
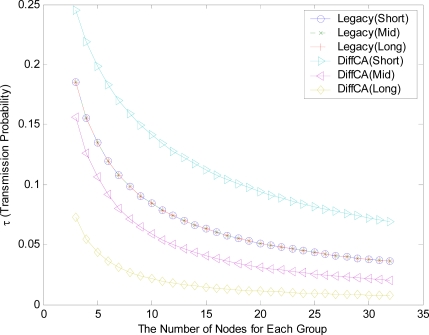
Comparison of transmission probabilities (τ).

**Figure 5.**
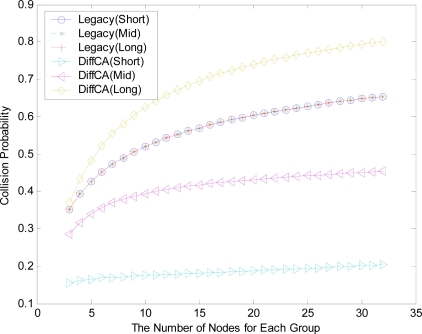
Comparison of collision probabilities.

Figure 4 shows the transmission probabilities, τ, for the three AGs. On the whole, as the number of nodes within each group increases, the contention level increases and the values of τ consequently decrease. In the legacy IEEE 802.15.4 system, all of the nodes in the network maintain the same probabilities, regardless of their packet size. In contrast, the probabilities of the nodes with the DiffCA algorithm vary according to their packet size. More specifically, AG 1 with the smallest packet size exhibits a higher probability than that of legacy systems, while AG 2 and AG 3 maintain relatively low transmission probabilities. Figure 5 shows the collision probabilities, which show a completely opposite tendency. Generally, as the number of nodes within a group increases, the collision of packets occurs more frequently, due to the increasing contention level. Moreover, AG 3 with the largest packet size exhibits a higher collision probability than the legacy systems. From the analytical results of Figures 4 and 5, it can easily be inferred that DiffCA preferentially guarantees the transmission of disadvantaged nodes, viz., nodes which generate small sized packets.

**Figure 6.**
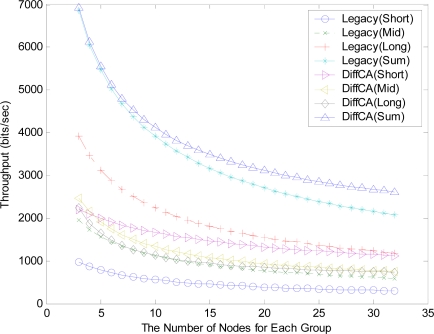
Comparison of saturation throughputs.

**Figure 7.**
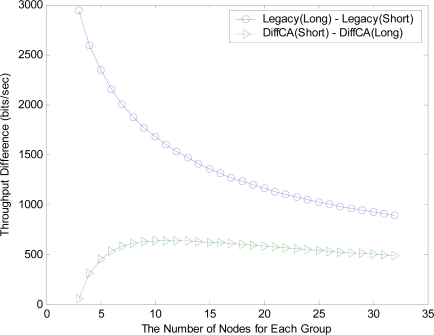
Comparison of throughput difference.

However, even if DiffCA grants nodes with small sized packets the first opportunity for channel access, it does not severely degrade the performance of the other nodes. Figure 6 shows the throughput for the three AGs. As shown in the figure, the nodes within the AGs using DiffCA exhibit similar performance in terms of the network throughput. On the contrary, in the legacy systems, the difference in the throughput between the node groups having different packet sizes is relatively large. This result is confirmed by the differences in the throughput in Figure 7.

In DiffCA, it is obvious that a node group with a large packet size has a low probability of accessing the channel. Nevertheless, it can retain the high performance since the quantitative traffic is sufficiently large. On the other hand, the performance of the node group with small sized packets cannot be significantly improved, even though the nodes in the group frequently access the channel. In the legacy IEEE 802.15.4 system, the node group with small sized packets exhibits very low throughput performance. Thus, it might be impossible for its WSN application to provide normal service.

Comprehensively, DiffCA can keep maintaining the local contention for advantaged nodes at a low level by distributing the timing of the transmission of the nodes in the network. As a result, the fairness between the application groups of the HSN in terms of the throughput performance can be significantly improved.

## 7. Conclusions

This paper presents DiffCA, which is a differentiated channel access scheme for IEEE 802.15.4-based heterogeneous sensor networks. DiffCA can provide different WSN application groups with enhanced fairness in terms of the network throughput by differentiating the opportunities for wireless medium access. A mathematical model based on the discrete-time Markov chain is provided for the purpose of analyzing the performance of the proposed scheme. A comparisons of the analytical and simulation results is given to verify the accuracy of the numerical model. The numerical results of several performance measures proved the effectiveness of DiffCA. We expect that the proposed scheme can provide guidance for the delicate tuning of the throughput performance among the WSN applications within one network.

## Figures and Tables

**Table 1.** Parameter set used in the numerical analysis and simulation.

The size of packet payload	AG 1 AG 2 AG 3	26 bytes 416 bytes 1,664 bytes 20 kbits/s 40 bits 200 bits 4 48 bits 20 symbols 1 Data bits in 860 MHz band
Channel bit rate ACK MAC header *macMaxCSMABackoffs* PHY header Unit backoff period Modulation symbol		

**Table 2.** Comparison of throughputs with various numbers of nodes.

**The number of nodes for each group**			**Analysis**				**Simulation**			
**Application Group**			**Application Group**			**Sum**	**Application Group**			**Sum**
**AG1**	**AG2**	**AG3**	**AG1**	**AG2**	**AG3**		**AG1**	**AG2**	**AG3**	

3	3	3	2,206.1	2,472.3	2,248.8	6,927.1	2,264.7	2,492.3	2,304.8	6,946.8
4	4	4	2,091.2	2,151.4	1,879.0	6,121.6	2,095.0	2,165.5	1,928.1	6,132.4
5	5	5	1,993.0	1,920.7	1,642.8	5,556.5	2,007.7	1,974.5	1,652.6	5,634.0
6	6	6	1,909.8	1,747.1	1,478.8	5,135.7	1,925.3	1,780.0	1,518.5	5,245.9
7	7	7	1,836.7	1,610.6	1,357.6	4,804.9	1,871.0	1,616.5	1,361.2	4,806.9
